# Retinoblastoma protein (Rb) links hypoxia to altered mechanical properties in cancer cells as measured by an optical tweezer

**DOI:** 10.1038/s41598-017-07947-6

**Published:** 2017-08-10

**Authors:** S. Khakshour, M. P. Labrecque, H. Esmaeilsabzali, F. J. S. Lee, M. E. Cox, E. J. Park, T. V. Beischlag

**Affiliations:** 1 School of Mechatronic Systems Engineering, Faculty of Applied Sciences, Simon Fraser University, Surrey, BC Canada; 2 Faculty of Health Sciences, Simon Fraser University, Burnaby, BC Canada; 3 Department of Urologic Sciences, The Vancouver Prostate Centre, University of British Columbia, Vancouver, British Columbia Canada

## Abstract

Hypoxia modulates actin organization via multiple pathways. Analyzing the effect of hypoxia on the biophysical properties of cancer cells is beneficial for studying modulatory signalling pathways by quantifying cytoskeleton rearrangements. We have characterized the biophysical properties of human LNCaP prostate cancer cells that occur in response to loss of the retinoblastoma protein (Rb) under hypoxic stress using an oscillating optical tweezer. Hypoxia and Rb-loss increased cell stiffness in a fashion that was dependent on activation of the extracellular signal-regulated kinase (ERK) and the protein kinase B (AKT)- mammalian target of rapamycin (MTOR) pathways. Pharmacological inhibition of MEK1/2, AKT or MTOR impeded hypoxia-inducible changes in the actin cytoskeleton and inhibited cell migration in Rb-deficient cells conditioned with hypoxia. These results suggest that loss of Rb in transformed hypoxic cancer cells affects MEK1/2-ERK/AKT-MTOR signalling and promotes motility. Thus, the mechanical characterization of cancer cells using an optical tweezer provides an additional technique for cancer diagnosis/prognosis and evaluating therapeutic performance.

## Introduction

In cancer and in particular, the tumour microenvironment, hypoxia is a pathological condition in which a significant region of the tumour is deprived of oxygen and is associated with increased risk of metastasis^1, 2^. Invasion and metastasis are complex and life threatening processes that transform anchored cells into mobile cells. Structural remodeling of the actin cytoskeleton is a critical component in most cancer cells for invasion and metastasis^3^. Recent studies on the effect of hypoxia on cell function revealed new information about the relationship between hypoxia and actin protein alterations that underlies the invasive cancer cell phenotype^4–7^. Modulation of actin organization under hypoxic conditions is complex and multiple pathways contribute to their alteration, such as Rho signalling pathways, the SCAP/SREBP1 pathway, MTOR phosphorylation pathways, p38 MAP kinase activation and HSP27 phosphorylation^8–12^. Elucidating different modulatory signalling pathways that alter actin organization and mediate the invasive cancer cell phenotype may prove a useful avenue for the development of novel anti-cancer therapeutic agents.

The hypoxic signal mediated by the HIF-1α-ARNT/HIF-1β transcriptional complex^13^ induces expression of genes associated with advanced stages of tumour growth and metastasis^14–16^. The retinoblastoma protein (Rb) is a tumour suppressor protein that is associated with the HIF-1α-ARNT-TRIP230 transcriptional complex and is a key regulator of the hypoxic response^13^. TRIP230 is an essential regulator of the hypoxic response^17^ and recruits Rb to HIF-1 target genes^18^. Subsequently, loss of Rb function results in biochemical changes that promote invasiveness in cancer cells^16, 18^.

Studying different signalling pathways that modulate actin organization under hypoxia is possible via analyzing the biophysical properties of cancer cells and quantifying cytoskeleton rearrangement^19^. Extracellular signal-related kinase (ERK) and protein kinase B (AKT) signalling pathways are important intracellular regulators of cell growth, proliferation, and malignant transformation^20^. Mitogen-activated protein kinase kinase -1 (MAPKK1 also known as MEK1) and MEK2 are tyrosine/threonine kinases that phosphorylate and activate ERK1/2 proteins^21^. The MEK1/2-ERK pathway plays an important role in actin organization and it can cause increases in cell motility and invasiveness by directly targeting actin proteins^22^. Moreover, AKT signalling can influence cell migration by modulating actin reorganization in the cell^23^. Additionally, MTOR signalling pathways control actin organization and regulates tumour cell invasion and motility^24^. MTOR is part of two distinct complexes. MTORC1 is known to regulate cellular proliferation and cell survival while MTORC2 modulates cytoskeleton reorganization through a direct effect on AKT^25^. Furthermore, the hypoxic signal mediated by the HIF-1α-ARNT transcriptional complex also causes expression of genes associated with tumour growth and metastasis. Gene ontogeny suggests that the Rb-HIF1 complex mediates the ERK1/2, AKT and NFκB signalling pathways, and therefore, perturbations in Rb expression may result in actin impairment and reorganization^16^.

Here we report cytoskeletal changes in human LNCaP prostate cancer cells that occur in response to loss of Rb under hypoxic stress using an oscillating optical tweezer (OT). The oscillating OT is an instrument that uses a highly focused laser beam to trap and oscillate a microbead attached to the cell cytoskeleton and therefore, exert a quantifiable force on the cell. The technical details of our setup have been described previously^26^. In summary, a continuous wave 3 W, Nd:YAG laser emitting light at a wavelength of 1064 nm was used with a Nikon TE2000 inverted microscope. A CCD camera and a CMOS camera were utilized to monitor the experimental process and to track the motion of the bead, respectively. LNCaP cells were selected as we have shown previously that hypoxia conditioned Rb-knockdown cells are capable of undergoing neuroendocrine differentiation (NED) and adopting an invasive phenotype^16^. Furthermore, interrogation of the LNCaP transcriptome identified the top up- and down-regulated genes sensitive to Rb-loss and hypoxia involved in metastasis and NED^16^. Nevertheless, we know little about actin structural changes and motile properties or the pathways that induce the invasive phenotype of Rb-deficient LNCaP cells. In order to quantify the cytoskeletal remodelling and stiffness of control and Rb-depleted LNCaP cells, movement of microbeads that bind to cell surface integrin receptors using an OT were measured and validated.

## Results

### Loss of Rb alters the mechanical properties of hypoxic LNCaP cells

**Figure 1:**
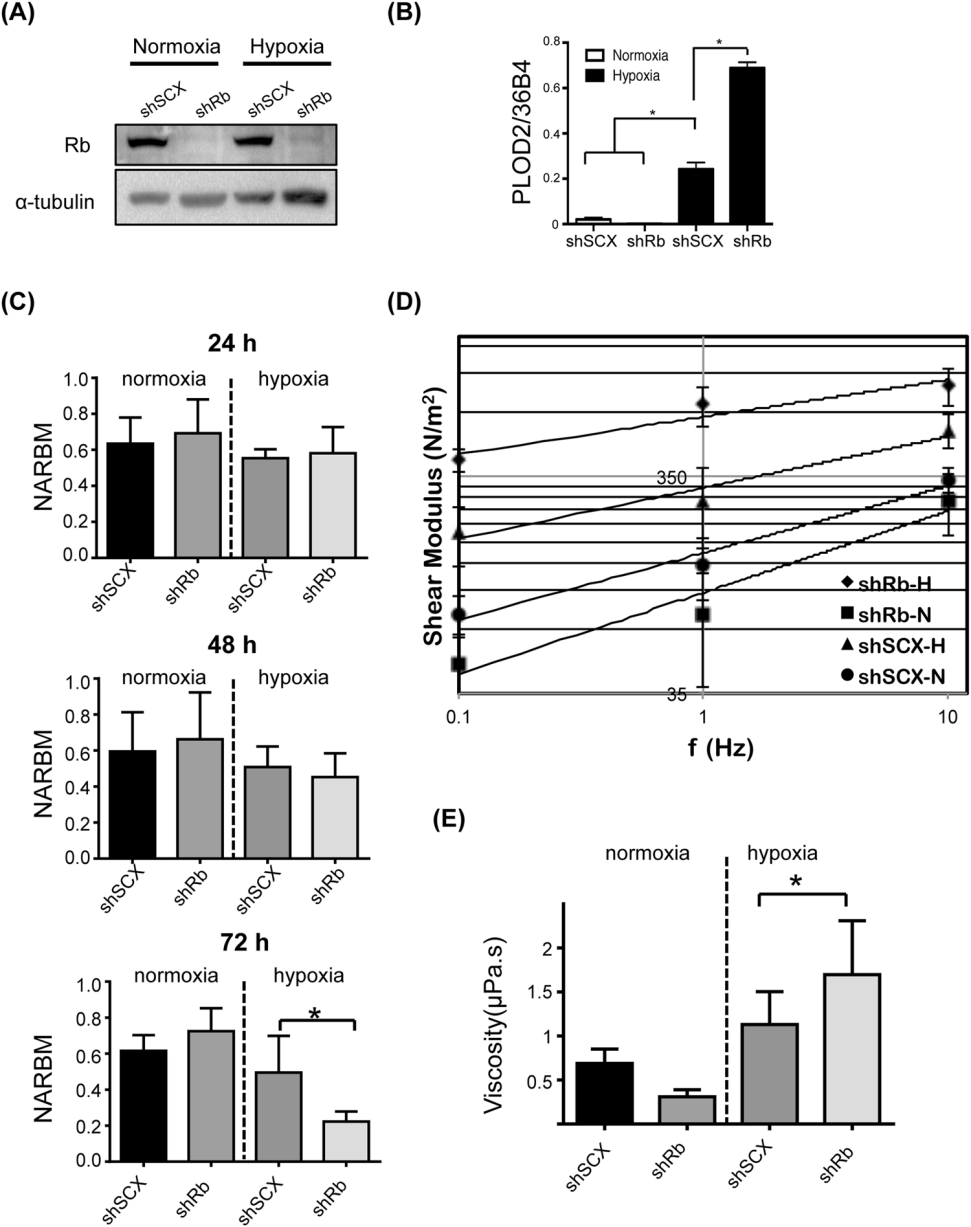
Exposure of prostate cancer cells to hypoxia causes dynamic changes in cell mechanical properties. (**A**) Immunoblot of Rb and α-tubulin protein expression in control shSCX and knockdown shRb LNCaP cells under hypoxic and normoxic conditions. Complete blot images are shown in Supplementary Information File 1, Figure S1. (**B**) PLOD2 mRNA accumulation in control shSCX and knockdown shRb LNCaP cells. Cells were maintained under normoxic conditions or 1% O_2_ for 24 h. Gene expression was determined by quantitative real-time PCR after isolation and reverse transcription of total RNA. PLOD2 expression was normalized to constitutively active 36B4 gene expression. (**C**) Variation in measured NARBM (Normalized Amplitude of Resultant Bead Movement) of shRb and shSCX LNCaP cells after 24 h, 48 h and 72 h of exposure to hypoxia or normoxia. (**D**) Variation in shear modulus after 72 h of exposure to hypoxia or normoxia at different frequencies of applied force (0.1, 1, and 10 Hz). (**E**) Variation in viscosity of shRb and shSCX LNCaP cells after 72 h of exposure to hypoxia or normoxia. In each experiment, at least 10 cells were examined for each group and each experiment was repeated five times. Error bars represent ± S.D. *p < 0.05.

To determine the effect of hypoxia and loss of Rb on the organization of the LNCaP cell cytoskeleton, we examined the **n**ormalized **a**mplitude of **r**esultant **b**ead **m**ovement (NARBM) in response to an OT applied force at a frequency of 1 Hz in short-hairpin-scrambled negative control (shSCX) and short-hairpin-Rb knockdown (shRb) cells under hypoxic and normoxic conditions (24, 48, 72 h). The shRb cells exhibited drastically reduced Rb protein compared to shSCX control cells and displayed a typical response to loss of Rb under hypoxic conditions as demonstrated by the exaggerated mRNA accumulation of the HIF1 target gene, procollagen-lysine,2-oxoglutarate 5-dioxygenase 2 (PLOD2) (Fig. 1A and B). Microbeads were coated with RGD and anchored to the cell cytoskeleton via integrins. Alterations in the measured movement of microbeads represents structural remodelling of cytoskeletal proteins^27–30^ and this has been linked to the membrane-actin cortex^31, 32^. Measuring the NARBM in response to the OT applied force demonstrated a time-dependent response in bead displacement that was significantly decreased in shRb cells exposed to 72 h of hypoxia compared to all other groups (Fig. 1C). These observations suggest that hypoxia promotes time dependent changes in cell structure that are measureable by tracking bead displacement in response to the OT’s applied force. Additionally, these results indicate that the hypoxia-inducible structural alterations in LNCaP cells observed by the RGD coated bead displacement was concomitant with actin redistribution. The bead displacement is limited by the quantity of integrin-linked actin tethered to the bead; therefore, alterations in actin distribution leads to concomitant changes in observed bead displacement. RGD is known to interact strongly with 8 distinct integrins - α_5_β_1_, α_8_β_1_, α_v_β_1_, α_v_β_3_, α_v_β_5_, α_v_β_6_, α_v_β_8_, α_IIB_β_3_^33^. RGD interacts only weakly with ITGA4 and examination of our LNCaP microarray data demonstrated that neither ITGA4 nor the mRNA levels of any of the other integrins were affected in response to hypoxia or loss of Rb^16^. Thus, changes in bead movement are likely not due to alterations in actin-associated integrin mRNA levels.

The measured bead displacements for each group of cells after exposure to 72 h of hypoxia or normoxia, along with the solution of the bead motion equation were used to identify viscoelastic properties of the cells^26^. The viscoelastic properties of the cell include shear modulus and viscosity coefficient. These parameters are measures of cell stiffness. More specifically, the shear modulus describes the elastic behavior of the cell cytoskeleton proteins and it is correlated with the distribution of the cell cytoskeletal proteins. The viscosity coefficient is associated with the viscose components of cells, such as cell cytoplasm. According to our results (Fig. 1D), shear modulus alterations for each group of cells were frequency dependent with a weak power-law dependency. Comparing the shear modulus at all applied frequencies of 0.1 Hz, 1 Hz, and 10 Hz, there exists a significant difference between hypoxia treated shRb cells and all other groups of cells. Both cell groups (shRb and shSCX) under hypoxia demonstrated an increase in shear modulus compared to normoxic cells, while the shRb group of cells showed the most significant increase in cell shear modulus at all frequencies (Fig. 1D). The viscosity coefficient response was not frequency dependent; however, there was a significant difference between the hypoxic shRb cells and all other treatments (Fig. 1E). In addition, the difference between normoxic shSCX cells and hypoxic shSCX cells was not significant. Finally, the viscosity coefficient was increased for the hypoxic conditioned cells compared to the cells cultured in normoxia. The power-law coefficient, which is an intrinsic cytoskeletal property, decreased under hypoxia for both shRb and shSCX cells compared to their normoxic conditioned cells. Thus, our results suggest that hypoxia alters the mechanical properties of Rb-deficient cells, which may be observed as measurable changes in the molecular organization of the cell cortex.

**Figure 2:**
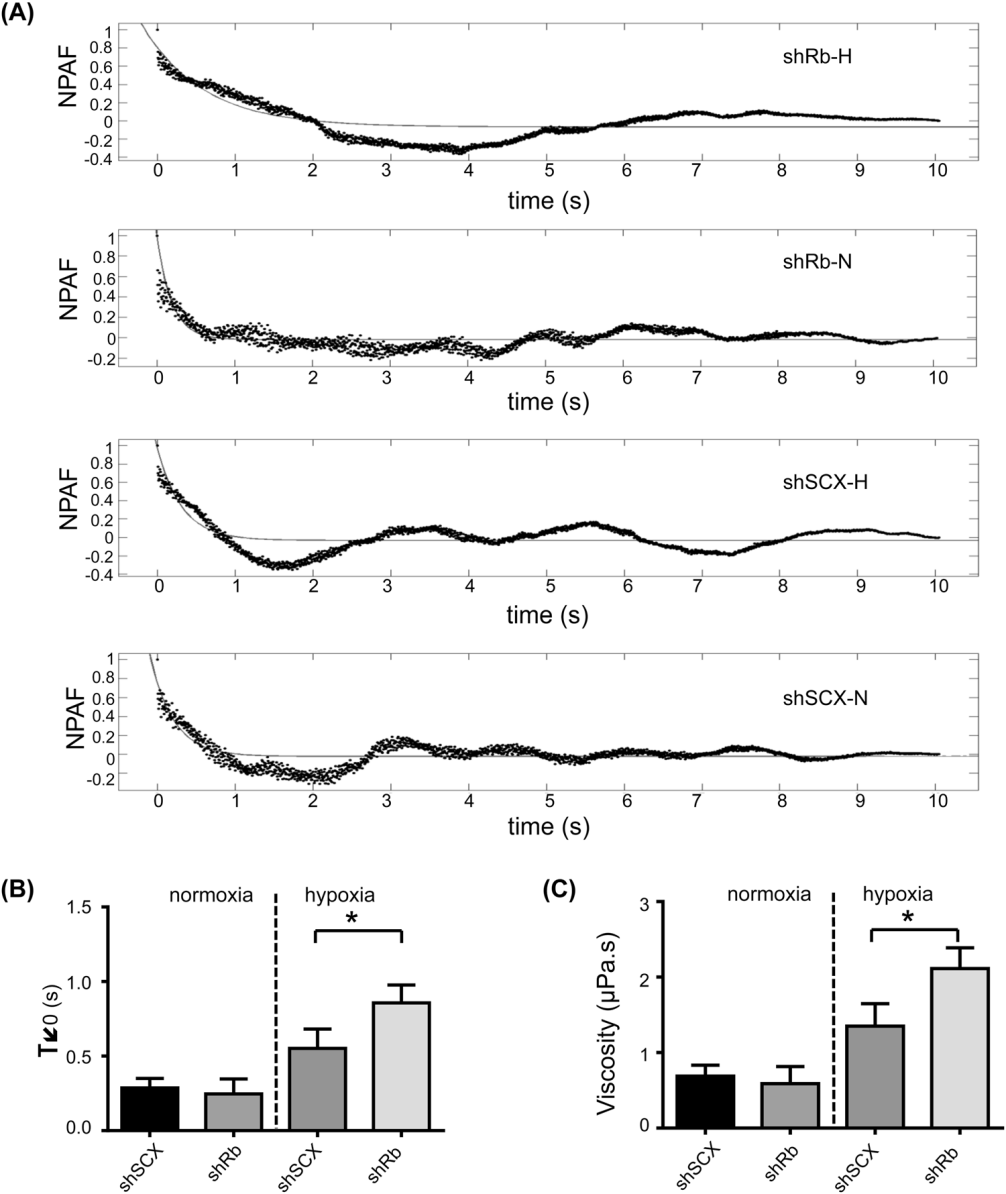
Exposure to hypoxia causes alterations in normalized position autocorrelation function (NPAF) in prostate cancer cells. (**A**) Normalized position autocorrelation function of beads fluctuations along with their exponential fit for shRb and shSCX LNCaP cells after 72 h of exposure to hypoxia or normoxia. (**B**) Relaxation time variation between different groups of cells (shRb, shSCX) after 72 h of hypoxia or normoxia. (**C**) Estimated viscosity from the measured relaxation time for different groups of cells (shRb, shSCX) after 72 h of hypoxia or normoxia. For **A**–**C** experiments were repeated 4 times (n = 4) and 10 cells were manipulated for each determination. Error bars represent ± S.D. *p < 0.05.

To further assess the effect of hypoxia and Rb-loss on the organization of the LNCaP cytoskeleton, we measured the relaxation time of control and Rb-depleted cells by monitoring the random movement of trapped beads anchored to the cell cytoskeleton^34^. More details about the derivation of relaxation time are presented in the data analysis section of the *Materials and Methods*. The **n**ormalized **p**osition **a**utocorrelation **f**unction (NPAF) of bead movement for the different groups of cells was plotted in Fig. 2A. The NPAF results were analyzed by fitting with exponential functions. According to these results, the time scale varied for different groups of cells; however, cells under hypoxic stress demonstrated a higher relaxation time compared to normoxic cells and hypoxic shRb cells had a significantly higher relaxation time compared to all other conditions (Fig. 2B). Moreover, the cell viscosity coefficient was derived based on the relaxation times and showed a significant increase between the hypoxia conditioned shRb cells and all other groups of cells (Fig. 2C). Accordingly, these findings are in agreement with our previous results and suggest that structural changes are occurring within the cell.

### The cell stiffness response and cytoskeleton alterations are dependent on ERK, AKT and MTOR

**Figure 3:**
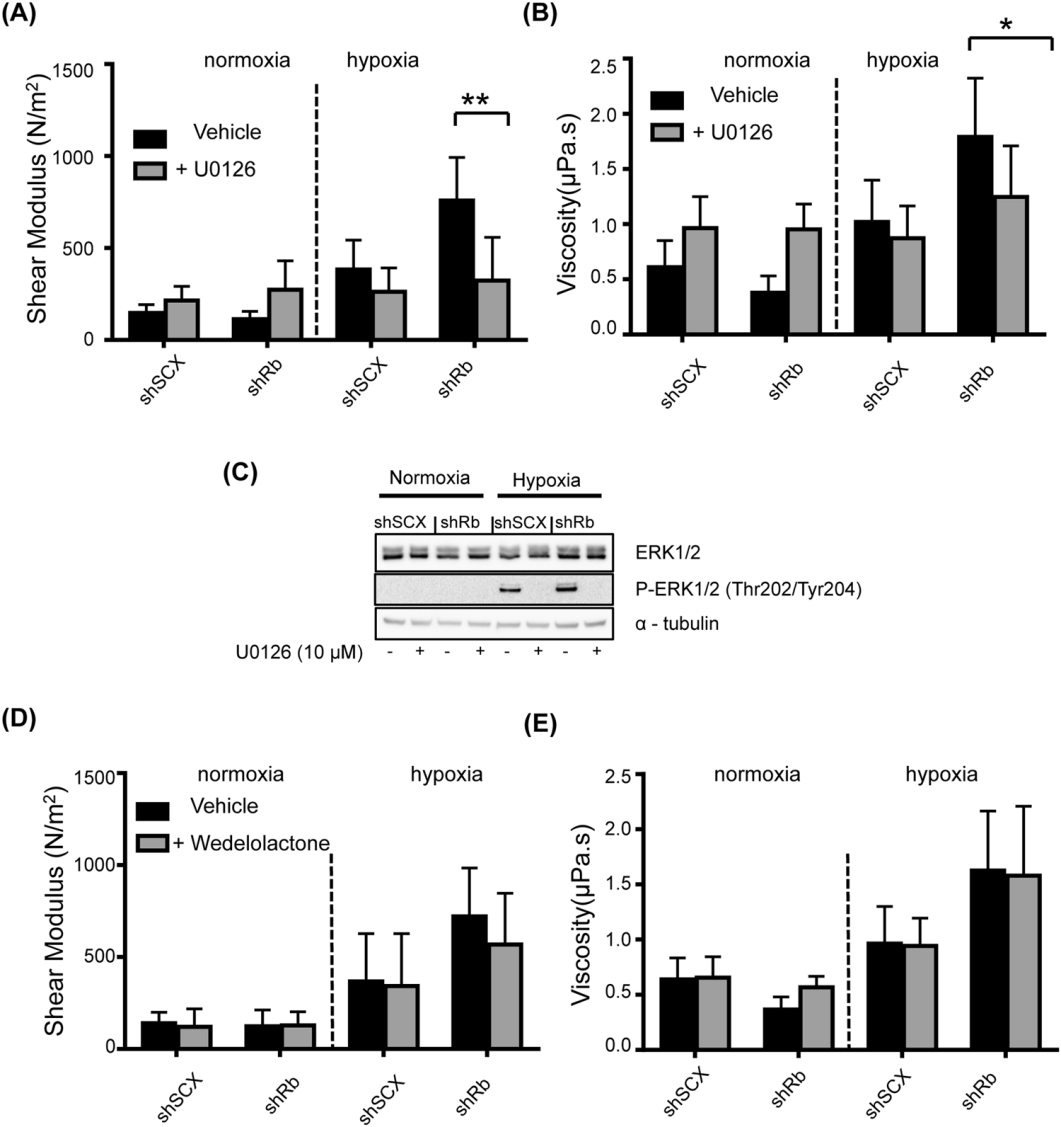
Inhibiting the MEK1/2-ERK pathway but not the NFκB pathway alters the mechanical properties of LNCaP cells. Variation in (**A**) shear modulus or (**B**) viscosity of shRb and shSCX LNCaP cells after exposure to 72 h of hypoxia or normoxia and treated with or without 10 μM U0126. (**C**) Immunoblots of LNCaP shRb and shSCX whole cell lysates after exposure to 72 h of hypoxia or normoxia and treated with or without 10 μM U0126. Blots were probed with antibodies against ERK1/2, phospho-ERK1/2 (Thr202/Tyr204) and α-tubulin. Samples were derived from the same experiment and blots were processed in parallel. Complete blot images are shown in Supplementary Information File 1, Figure S1. Variation in (**D**) shear modulus or (**E**) viscosity of shRb and shSCX LNCaP cells after exposure to 72 h of hypoxia or normoxia and treated with or without 15 μM wedelolactone. For **A**, **B**, **D** and **E**, experiments were conducted in triplicate (n = 3) and ten cells were examined in each experiment. Error bars represent ± S.D. *p < 0.05. **p < 0.001.

Next we wanted to determine the pathway responsible for the observed increase in cell stiffness. Gene ontology analysis of the LNCaP transcriptome identified the MEK1/2-ERK1/2 pathway as a likely regulator of hypoxia-inducible cell motility in Rb-knockdown cells^16^. In order to determine if the MEK1/2-ERK1/2 pathway is responsible for changes in actin distribution, shRNA LNCaP cells were treated with vehicle or 10 μM of the MEK1/2 inhibitor U0126 and then conditioned with either normoxia or hypoxia. Cell mechanical properties were determined by applying time varying force on beads anchored to the cytoskeleton, employing the OT and measuring bead displacement. The Rb-depleted (shRb) cells under hypoxic stress showed a significant increase in shear modulus compared to the scrambled negative control (shSCX) cells (Fig. 3A). While there were no significant differences between shSCX or shRb cells maintained under normoxia and treated with or without U0126, hypoxic shRb cells treated with U0126 demonstrated a significantly lower shear modulus compared to untreated hypoxic shRb cells (Fig. 3A). Likewise, hypoxia conditioned shRb cells demonstrated a significant increase in cell viscosity compared to normoxic cells and hypoxic shSCX cells, however, this effect disappeared with the addition of 10 μM U0126 (Fig. 3B). Immunoblots of whole cell lysates determined that both shSCX and shRb LNCaP cells conditioned with hypoxia expressed elevated levels of phosphorylated ERK1/2 protein, a bona fide marker of the hypoxic response^35, 36^. Importantly, addition of 10 μM U0126 blocked ERK1/2 phosphorylation (Fig. 3C). These results suggest that hypoxia increased cell stiffness in Rb knockdown cells in an ERK-dependent fashion. Inhibition of the MEK1/2-ERK pathway using U0126 inhibited the alterations in actin structure, which was observed as decreased cell shear modulus. Furthermore, we studied the effect of the NFκB pathway on actin disruption by treating cells with 15 μM of wedelolactone; an NFκB inhibitor that blocks the phosphorylation and degradation of IκBα. The mechanical responses of the cells were again calculated upon applying time varying force on beads adhered to LNCaP cells using the OT and measuring bead displacement. The result showed no significant differences between cells treated with or without wedelolactone (Fig. 3D and E). Thus, NFκB does not play a role in altered cell stiffness of Rb knockdown LNCaP cells in response to hypoxia.

**Figure 4:**
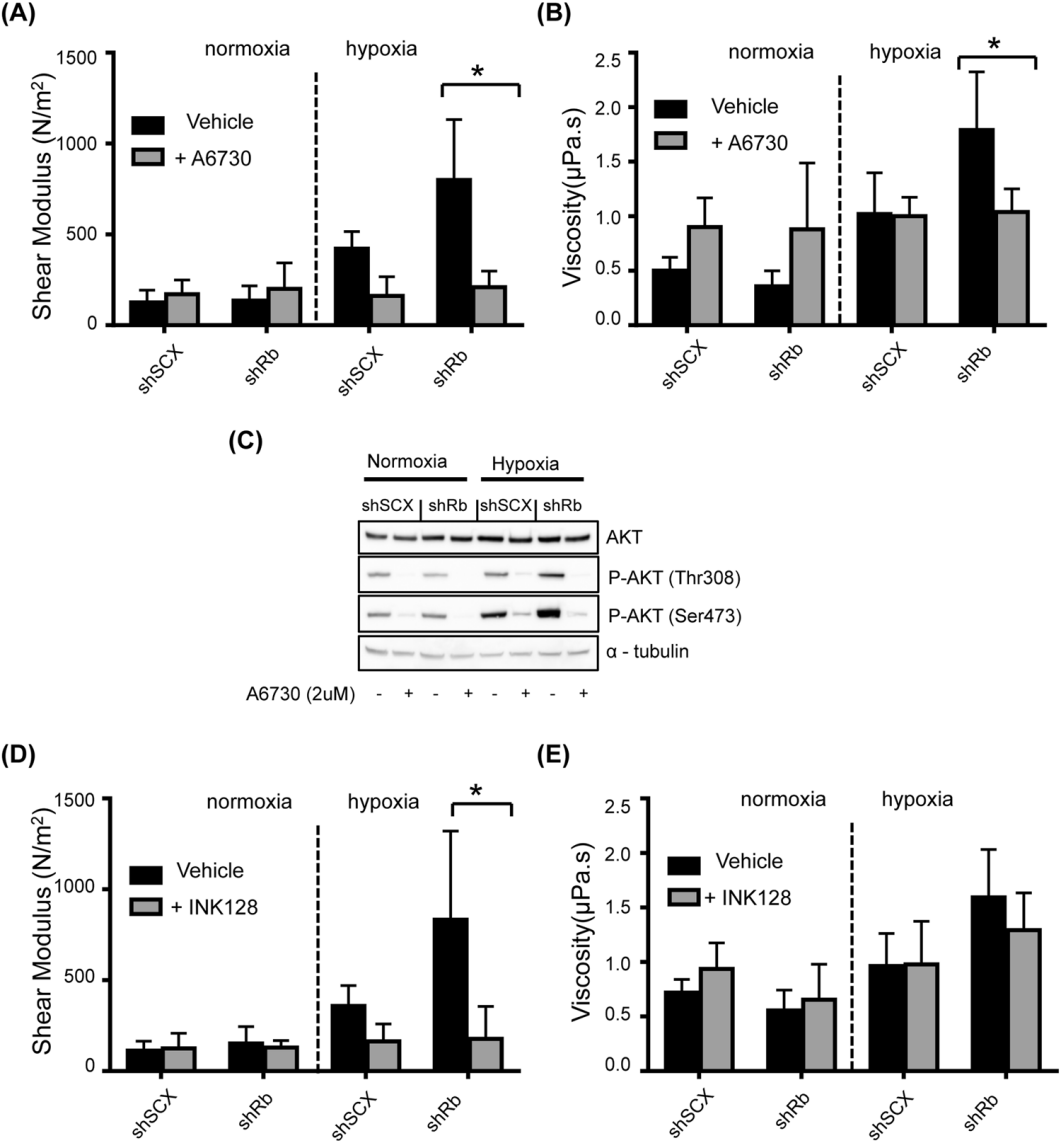
Inhibiting either the AKT or MTOR pathways alters the mechanical properties of LNCaP cells. Variation in (**A**) shear modulus or (**B**) viscosity of shRb and shSCX LNCaP cells after exposure to 72 h of hypoxia or normoxia and treated with or without 2 μM A6730. (**C**) Immunoblots of LNCaP shRb and shSCX whole cell lysates after exposure to 72 h of hypoxia or normoxia and treated with or without 2 μM A6730. Blots were probed with antibodies against AKT, phospho-AKT (Thr308), phospho-AKT (Ser473) and α-tubulin. Samples were derived from the same experiment and blots were processed in parallel. Complete blot images are shown in Supplementary Information File 1, Figure S1. Variation in (**D**) shear modulus or (**E**) viscosity of shRb and shSCX LNCaP cells after exposure to 72 h of hypoxia or normoxia and treated with or without 10 nM INK128. For **A**, **B**, **D** and **E**, experiments were conducted in triplicate (n = 3) and ten cells were examined in each experiment. Error bars represent ± S.D. *p < 0.05.

In order to further characterize our results, we examined the role of AKT, a regulator of actin organization that works in parallel and cross-talks with the ERK1/2 signalling pathway^37, 38^. We maintained shRNA LNCaP cells in either normoxia or hypoxia and treated cells with or without 2 μM A6730, an inhibitor of AKT. Interestingly, we found that inhibiting AKT phosphorylation produced alterations in cell shear modulus similar to inhibiting ERK phosphorylation (Fig. 4A and B). Immunoblots of total cell lysates using an affinity purified antibody specific for total and phosphorylated AKT demonstrated that shRb cells cultured in hypoxic conditions had higher levels of phosphorylated AKT compared to all other groups of cells and that AKT phosphorylation was prevented by treatment with A6730 (Fig. 4C). Moreover, we determined the role that MTOR might play in this phenomenon. MTOR is a downstream effector of the AKT and MEK-ERK pathways and a regulator of actin organization^39, 40^. The shear modulus and viscosity of LNCaP cells treated with or without 10 nM INK128, a specific inhibitor of MTOR, and incubated under normoxic or hypoxic conditions were measured (Fig. 4D and E). We observed a significant increase in the shear modulus of untreated hypoxic Rb knockdown cells. This condition was reversed with INK128 treatment, which is in agreement with our observations using A6730 and U0126. Thus, our results demonstrate that both MEK-ERK-MTOR and AKT-MTOR signalling pathways are responsible for the changes in the mechanical properties we observed in Rb-depleted hypoxic LNCaP cells.

**Figure 5:**
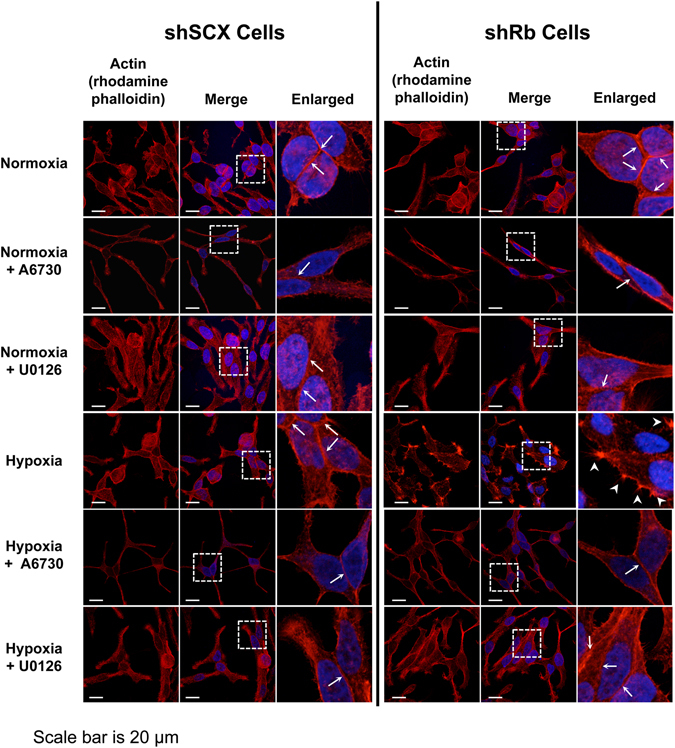
Immunofluorescence microscopy of actin in LNCaP cells. shRb and shSCX LNCaP cells were exposed to 72 h of hypoxia or normoxia and treated with or without 10 µM U0126 or 2 µM A6730. Fixed cells were treated with DAPI and TRITC-labelled rhodamine phalloidin and were imaged with a Zeiss LSM-780 confocal microscope. Phalloidin only images are represented in the left panels, merged images are in the middle panels and enlarged merged images are represented in the right panels. Filopodia-like processes are highlighted with large white arrow heads and cell:cell actin borders are shown with white arrows. Each experiment was repeated four times.

**Figure 6:**
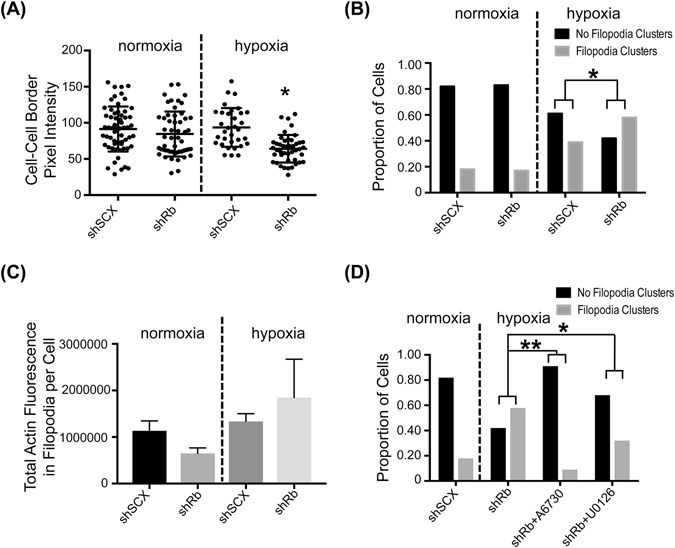
Rb-loss and hypoxia alter the LNCaP actin cytoskeleton. (**A**) Scatter plot of pixel intensities measured along cell-cell boundaries. A double-blind analysis of cell-cell boundaries from normoxic shSCX (n = 60) and shRb (n = 53) and hypoxic shSCX (n = 33) and shRb (n = 44) conditions was conducted using ImageJ software. Horizontal black bars represent mean pixel intensities ± S.D., differences between means were determined by a Kruskall-Wallis with Dunn’s multiple comparisons test, *p-value < 0.05. (**B**) Proportion of cells categorized as having either no filopodia-like clusters (black bars) or filopodia-like clusters (grey bars). Cells from normoxic shSCX (n = 141) and shRb (n = 134) and hypoxic shSCX (n = 95) and shRb (n = 123) conditions were categorized through a double-blind analysis of phalloidin staining. Differences between populations were determined with a Fisher’s exact test, *p-value = 0.0065. (**C**) Total actin fluorescence in filopodia clusters per cell was determined by multiplying the phalloidin staining intensity by the area of each measured filopodia cluster. Phalloidin staining intensity and area of filopodia clusters were determined through a double-blind analysis of confocal images using ImageJ software. Data represents means ± S.E.M. (**D**) Proportion of hypoxia conditioned shRb cells categorized as having either no filopodia-like clusters (black bars) or filopodia-like clusters (grey bars) after treatment with vehicle (n = 123), 2 µM A6730 (n = 78) or 10 µM U0126 (n = 103). Cells were categorized through a double-blind analysis of phalloidin staining. The normoxic shSCX cells (n = 141) are shown as a negative control. Differences between populations were determined with a Fisher’s exact test, *p-value = 0.0002, **p-value < 0.0001.

As previously stated, changes in **n**ormalized **a**mplitude of **r**esultant **b**ead **m**ovement (NARBM) in response to the OT applied force is an indication of cytoskeletal remodelling^27–30^. However, we wanted to confirm that the changes in cell viscoelastic properties that we observed in Rb-deficient LNCaP cells led to actin reorganization. Thus, we performed immunofluorescence microscopy using rhodamine phalloidin as an actin probe on shRNA LNCaP cells conditioned with either hypoxia or normoxia and treated with or without U0126 or A6730 (Fig. 5). Both normoxic shSCX and normoxic shRb cells displayed a typical LNCaP phenotype with an elongated cell structure, a rather even distribution of actin fluorescence and the presence of well-defined cell-cell boundaries (Fig. 5, 1^st^ row, thin white arrows point to cell-cell boundaries). Rb-knockdown cells exposed to hypoxia demonstrated altered actin distribution compared to normoxic cells and profound morphological changes were observed (Fig. 5, right-hand panels, fourth row). Actin fluorescence in shRb hypoxic cells seemed to diminish along cell-cell boundaries and concentrate in distinct filopodia-like clusters along the cell periphery (Fig. 5, right-hand panels, fourth row, large white arrowheads point to filopodia-like clusters) a common feature of metastatic transformation^41^. To a lesser degree, some hypoxic control cells seemed to also undergo distinct morphological changes and some actin redistribution but the majority of cells retained their elongated phenotype with rather more distinct and uniform actin distribution along the cell border (Fig. 5, left-hand panels, fourth row, thin white arrows point to cell-cell boundaries). In addition, a double-blind analysis using ImageJ software to characterize cell-cell boundaries and filopodia-like processes determined significant phenotypic differences between hypoxic shRb cells and all other conditions (Table 1). Specifically, a Kruskall-Wallis analysis with Dunn’s multiple comparisons test (alpha = 0.05; P-value < 0.05) determined that the average pixel intensity in cell-cell boundaries was significantly lower in hypoxic shRb cells (64.16 ± 19.10) compared to hypoxic shSCX cells (93.57 ± 26.78) and to normoxic shSCX (91.44 ± 31.19) and shRb cells (84.54 ± 31.11) suggesting a redistribution of actin away from cell-cell boundaries in hypoxic shRb cells (Fig. 6A). Importantly, a two-sided Fisher’s exact test determined that the proportion of cells with filopodia-like clusters was significantly different between hypoxic shRb cells and hypoxic shSCX cells, 0.59 and 0.38 respectively with P-value = 0.0065 (Fig. 6B). Furthermore, the proportion of cells with filopodia-like processes was also significantly different between hypoxic shRb cells and normoxic controls with P-value < 0.0001. Although the proportion of hypoxic shRb cells displaying filopodia-like clusters was significantly different compared to the other treatments, quantifying the number of filopodia and average amount of actin in the filopodia-like clusters was too difficult to parse out as the areas of defined clusters varied widely within the cell populations making the standard deviations of quantified measurements too large for statistical significance (Fig. 6C, Table 1 and Supplementary Dataset Files 1 and 2). Nevertheless, our morphological analysis supports the results we obtained from our mechanical characterization studies and suggests that Rb-loss significantly changes the organization of actin in hypoxic LNCaP cells. Raw values for all our measurements can be found in Supplementary Dataset File 1 and summaries of our analyses can be found in Supplementary Dataset File 2.

**Table 1:** Summary of the double blind analysis of actin fluorescence.

Treatment	Cell-Cell Boundaries			Filopodia Clusters			Mean Total Actin Fluorescence in Filopodia per Cell (±S.D.)
	Total Cell Boundaries	Mean Pixel Intensity (±S.D.)	P-value^a^ (vs shRb H)	Total Cells	Proportion of cells with filopodia	P-value^b^ (vs shRb H)	
shSCX N	60	91.44 ± 31.19	<0.05	141	0.18	<0.0001	1133617.84 ± 1064723.26
shRb N	53	84.54 ± 31.11	<0.05	134	0.17	<0.0001	649978.37 ± 528087.88
shSCX H	33	93.57 ± 26.78	<0.05	95	0.39	0.0065	1333473.20 ± 1022056.79
shRb H	44	64.16 ± 19.10	N/A	123	0.58	N/A	1848934.20 ± 6925370.43

^a^P-values were determined using the Kruskal-Wallis with Dunn’s multiple comparisons test.

^b^P-values were determined using the Fisher’s exact test.

**Figure 7:**
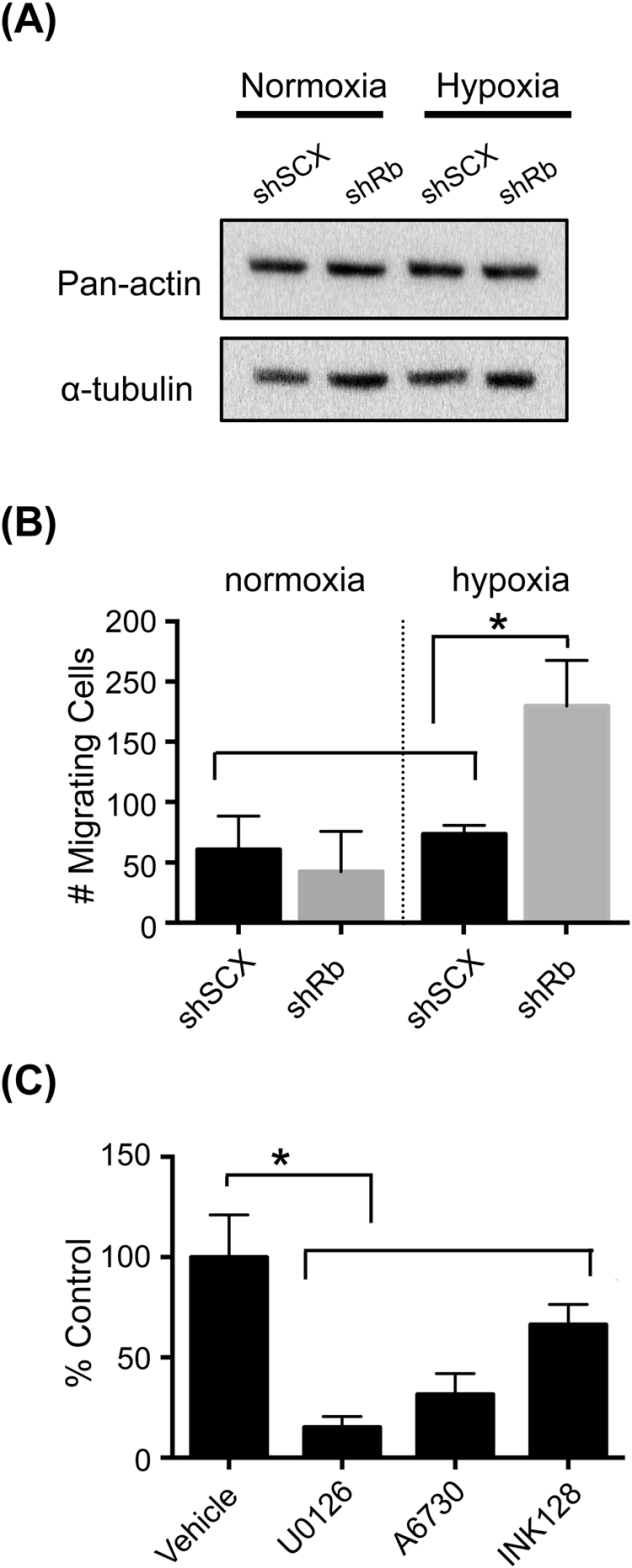
Exposure to hypoxia increased the total number of migrating shRb LNCaP cells. (**A**) Immunoblot of LNCaP shRb and shSCX whole cell lysates after exposure to 72 h of hypoxia or normoxia and probing with primary antibodies to pan-actin or α-tubulin. Samples were derived from the same experiment and blots were processed in parallel. Complete blot images are shown in Supplementary Information File 1, Figure S1. (**B**) Total number of migrating cells for shRb and shSCX LNCaP cells after exposure to hypoxia or normoxia. (**C**) Total number of migrating cells for shRb LNCaP cells conditioned with hypoxia and treated with DMSO, 10 μM U0126, 2 μM A6730 or 10 nM INK128. Experiments were performed in triplicate. Error bars represent ± S.D. *p < 0.05.

Interestingly, pre-treatment of cells with either 10 μM U0126 or 2 μM A6730 impeded the hypoxia-inducible alterations in cellular morphology and redistribution of actin fluorescence in the shRb cells (Fig. 5). Indeed, treatment with A6730 and to a lesser extent with U0126 significantly reduced the proportion of cells exhibiting filopodia-like clusters in hypoxic shRb cells and inhibited the morphological changes promoted by hypoxia and Rb-loss (Fig. 6D and Supplementary Dataset Files 1 and 2). Finally, immunoblots of total cell lysates revealed that the dramatic cytoskeletal changes in shRb cells conditioned with hypoxia was not due to altered actin levels (Fig. 7A). Taken together, these results demonstrate that both the AKT and MEK1/2-ERK1/2 pathways play a critical role in hypoxia-inducible alterations of the actin cytoskeleton in Rb-deficient LNCaP cells. In addition, changes in cell stiffness response measured by the OT correlate with changes in actin remodelling.

### Hypoxia inducible migration is dependent on ERK, AKT and MTOR

A growing body of evidence in the literature^23, 42^ suggest that the AKT and ERK signalling pathways control cell migration. Furthermore, gene ontology analysis^16^ suggests that Rb is linked to this process in hypoxic LNCaP cells. To determine if actin reorganization correlates with increased cell migration, we conditioned shRNA LNCaP cells with hypoxia or normoxia and used a cell migration assay to measure the total number of migrating cells. Indeed, shRb LNCaP cells exposed to hypoxia migrated at a significantly higher rate compared to scrambled negative controls and to normoxic shRb cells (Fig. 7B). Additionally, treatment with 10 μM U0126, 2 μM A6730 or 10 nM INK128 significantly impeded hypoxia-inducible migration in shRb LNCaP cells compared to the untreated control (Fig. 7C). Taken together, these data support our hypothesis that the ERK1/2 and AKT pathways may crosstalk through MTOR to control migration and invasion in hypoxic prostate cancer cells lacking Rb. Moreover, in our study, the cell migration data correlates with the OT data. In the future, we will investigate if OT measurements could be used to predict migratory cell phenotypes in a more general fashion.

## Discussion

In this study we examined the hypoxia-inducible changes in biophysical properties of control and Rb-knockdown LNCaP prostate cancer cells using an oscillating OT. Our results indicate that loss of Rb protein in prostate cancer cells under hypoxic conditions increases migration and alters cytoskeletal protein dynamics by activating the downstream signalling modules. The over-activity of either the ERK1/2 or AKT pathways facilitates alterations in the cytoskeleton that promote motility and result in structural changes and actin protein redistribution without altering the level of total actin in the cell or by increasing mRNA levels of actin-associated integrins. These alterations correlated with changes in cell stiffness that were estimated by measuring the resultant movement of beads (NARBM) anchored to the cytoskeleton. Changes in bead movement correspond to changes in cell stiffness and in turn, actin organization, where decreased movement is likely a result of redistribution and stabilization of actin and/or an increase in cell tethering forces at focal adhesion sites either on the bead or on the cell substrate^32^. This increase in cortical stiffness at focal adhesion sites might translate into increased migratory capacity of the cell via a leader-bleb mechanism^43^. Furthermore, inhibition of either the ERK1/2 or AKT-MTOR pathways prevented actin reorganization that may contribute to cancer cell transformation and motility. In order to analyze alterations in the mechanical properties of control and Rb-knockdown LNCaP cells, resultant bead movements were measured under hypoxic and normoxic conditions using an oscillating OT^26^. The results show time dependent changes in bead movement for cells (shSCX and shRb) cultured under hypoxia for 72 h. In addition, increased cell migration in hypoxic shRb LNCaP cells suggests that there is a correlation between increased cell migration and actin reorganization.

Activation of the ERK1/2 pathway has a profound effect on actin disruption in many cell types^44–48^; however, these findings were mainly based on structural evidence. Consistent with these structural observations, we measured and analyzed cell mechanical properties and investigated the role of the ERK pathway on actin reorganization. Based on our results, the parallel activation of both the MEK1/2-ERK and AKT pathways are responsible for actin reorganization and morphological changes in Rb-deficient hypoxic cancer cell lines. Inhibition of either the MEK1/2-ERK or AKT pathways using U0126 or A6730, respectively prevented or impeded the development of actin-containing filopodia and inhibition of other hypoxia-induced morphological changes in Rb-ablated LNCaP cells (Fig. 5). Moreover, we measured alterations in cell mechanical properties after inhibiting MTOR, the downstream effector of the AKT and MEK-ERK pathways. Inhibition of the MTOR pathway phenocopied inhibition of the MEK-ERK or AKT pathways, suggesting that MTOR is a downstream effector of both the MEK-ERK and AKT pathways for actin reorganization in Rb-depleted hypoxic cancer cell lines. Additionally, other HIF1-regulated pathways may be sensitive to loss of Rb and contribute to this phenotype. Activation of focal adhesion kinase and phosphorylation of paxillin correlate with hypoxia-inducible migration of C6 glioma cells^49^. Up-regulation of vimentin intermediary filaments has been implicated in epithelial-to-mesenchymal transition and increased migratory capacity in lung cancer cells in an AKT-dependent fashion^50^. However, hypoxia-induced reorganization of filamentous actin is concomitant with vimentin condensation in an ERK-dependent fashion^51^. The relative contribution of actin and vimentin reorganization to the migratory capacity of the cell should be resolved using RNA knockdown technologies.

**Figure 8:**
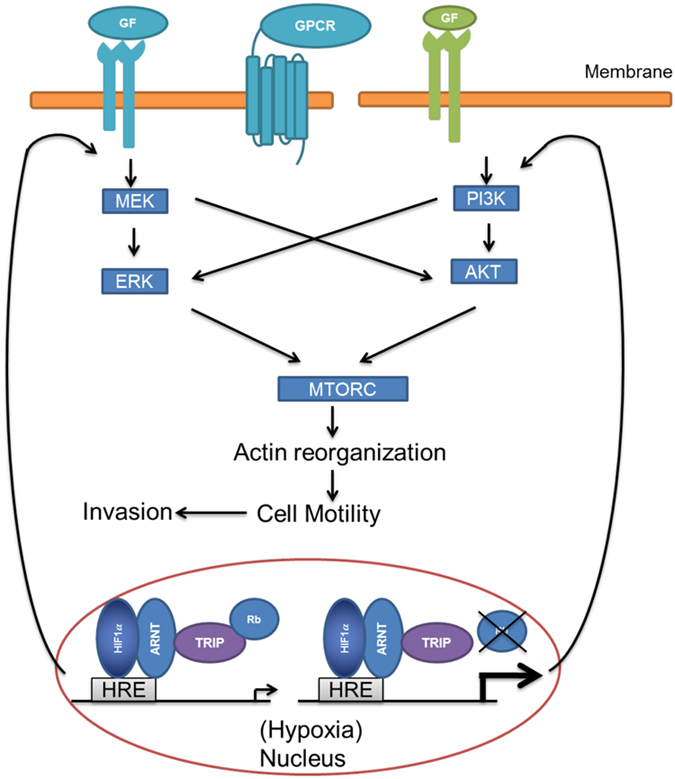
Signalling pathways responsible for changes in actin cytoskeleton. Loss of Rb in hypoxic prostate cancer cells increases actin reorganization and migration through ERK-AKT-MTOR signalling. GF: growth factor; GPCR: G protein coupled receptor; HRE: hypoxia response element; ARNT: aryl hydrocarbon receptor nuclear translocator; TRIP230: thyroid hormone receptor/retinoblastoma-interacting protein 230.

The evidence presented here suggests that loss of Rb in hypoxic prostate cancer cells increases actin reorganization and migration through ERK-AKT-MTOR signalling (Fig. 8). Several studies have demonstrated crosstalk between PI3K-AKT-MTOR and MEK-ERK signalling pathways^52–54^. However, depending on the stimulus, the pathways may have cooperative, inhibitory or separate biological functions. For example, MEK1, MEK2 and MEK5 inhibitors enhance AKT activation possibly involving phosphorylation of GAB1 induced by ERK2 and inhibition of recruitment of PI3K to the EGF receptor^55^. Alternatively, it has been reported that AKT can phosphorylate inhibitory sites in Raf, and consequently down regulate ERK1/2^56^. Conversely, cross-activation has also been reported. In this instance, Ras-ERK regulates PI3K, and MTORC1 which results in cross activation of PI3K-MTORC1^57^. Furthermore, the ERK1/2 and AKT promote MTORC1 activity by inhibiting TSC1/2’s GAP function^58^.

Previously, we determined that loss of Rb dysregulates HIF1-mediated transcriptional responses^18^ and that Rb-loss in conjunction with hypoxia leads to the acquisition of a more invasive and neuroendocrine phenotype in prostate cancer cells^16^. We identified several putative upstream regulators of ERK1/2 and AKT-MTOR signalling that may contribute to prostate cancer cell transformation, such as CXCR4, HTR5A and KISS1 receptor (KISS1R)^16^. Furthermore, loss of Rb expression and hypoxia sensitized prostate cancer cells to kisspeptin-10, a potent KISS1R agonist^16^. Although KISS1/KISS1R interactions have been linked to both pro- and anti-metastatic processes, KISS1R expression in breast cancer cells increases cell invasion and metastasis^59, 60^. Additionally, KISS1R signalling activates ERK1/2 through β-arrestin^61^ and stimulates invadopodia formation in triple negative breast cancer via β-arrestin and ERK1/2-dependent mechanisms^62^. The KISS1R-ERK1/2-invadopodia paradigm requires further investigation in prostate cancer, however, we speculate that some Rb-negative prostate cancer cells under hypoxic stress may become more invasive in a similar fashion.

Magnetic twisting cytometry (MTC) and atomic force microscopy have been employed previously to measure alterations in the mechanical properties of cancer cells in order to characterize the pathways responsible for actin reorganization^19, 63–65^. Stiffness of pulmonary microvascular endothelial cells exposed to hypoxia was measured using MTC. These investigators determined that hypoxia increased cell stiffness and therefore regulates endothelial cell contraction^19^. Cytoskeletal dynamics were measured in skin, bladder, prostate and kidney cancer cell lines. These results suggested that metastatic cancer cells exhibit increased localized cortical cell stiffness. This increased cell stiffness is required to facilitate the vascular invasion^63–65^. The mechanical response to the anticancer drugs colchicine or cytarabine exhibited by cervical and liver carcinoma cells were measured and these results demonstrated a correlation between the degree of mechanical changes in cells and the drug dosages^63–65^. Thus, employing MTC and atomic force microscopy could provide useful insights into cancer cell cytoskeletal dynamics, alterations and metastatic processes. Comparing the OT to other technologies, we find that its main advantage is being a noninvasive technique that can apply a uniform stress on cells that could aid in measuring cellular mechanical alterations more accurately. Our results demonstrate that the OT could be used to measure the mechanical properties of cells that correlate with cell migration. Thus, this technique could be used to evaluate drug candidates that target cell migration for their ability to alter those mechanical properties.

Our study suggests, that factors which regulate the hypoxic signals and thereby lead to the untoward activation of the ERK and AKT pathways, might be suitable therapeutic targets for prostate cancer metastasis, namely the HIF1 multi-protein transcriptional complex. Clinical trials of AKT and ERK inhibitors have demonstrated unacceptable toxicities^66, 67^ suggesting that targeting deregulated upstream factors may be more selective in treating prostate cancers. In conclusion, these results highlight the utility of the optical tweezer in characterizing the mechanical properties of cells, which might aid in identifying transformed cancer cells.

## Materials and Methods

### Materials and reagents

RGD peptide, wedelolactone, U0126 and A6730 were purchased from Sigma-Aldrich (ON, Canada). The microbeads were purchased from Bangs Laboratory (ON, Canada).

### Cell culture and exposure to hypoxia

The production of control shSCX- and knockdown shRb-LNCaP cells was described previously^16^. Cells were maintained in RPMI 1640 medium with L-Glutamine (BioWhittaker, Lonza), supplemented with 10% FBS and 1% 100 units/ml potassium penicillin- 100 μg/ml streptomycin sulphate. For hypoxia treatment, cells were placed into a humidified, hypoxia incubation chamber and maintained in 5% CO_2_, and 1% O_2_ at 37 °C. For normoxia treatment, cells were kept in a humidified incubation chamber and maintained at 37 °C with 5% CO_2_, and 20% O_2_.

### Quantitative Real-Time PCR

LNCaP cells were incubated under hypoxic conditions (1% O_2_) for 24 h in a humidified CO_2_ incubator. The mRNA levels of PLOD2 and 36B4 were determined using quantitative real-time PCR. The primer pairs for PLOD2 and 36B4 were described previously^18, 68^. Total RNA was isolated using TRI reagent (Sigma, Cat. No. T9424–200ML) according to the manufacturer’s protocol. Reverse transcription was performed using High Capacity cDNA Reverse Transcription Kit (Applied Biosystems, Part No.4368814) according to the manufacturer’s protocol. A total of 2 µg of RNA was used in a 20 µL reaction amplified by cycling between 25 °C for 5 min, 37 °C for 120 min, and 85 °C for 5 min (Veriti 96 Well Thermal Cycler, Applied Biosystems). For each experiment, a sample that was both infected with viral Rb-specific shRNA and pre-conditioned with hypoxia was used to generate a relative standard curve in which the sample was diluted 1:10 in five serial dilutions resulting in dilutions of 1:10, 1:100, 1:1,000, 1:10,000, and 1:100,000 whereas the samples were diluted 1:30; the analysis was done using StepOnePlus System (Applied Biosystems).

### Antibodies and immunoblotting

LNCaP cells were maintained under normal oxygen (normoxia, 20% O_2_) or incubated under hypoxic conditions (1% O_2_) for 24 h. Cells were then treated with either vehicle (DMSO) or drug (2 μM A6730 or 10 μM U0126) and then left at normoxia or hypoxia for a further 48 h. Cells were harvested and the protein concentration estimated by the RC DC Protein Assay (Bio-Rad). Equal amounts of total protein from the samples were resolved on a SDS-acrylamide gel then transferred to polyvinylidene fluoride (PVDF) membrane. Membranes were incubated with diluted primary antibodies in 5% w/v skim milk powder, 1X TBS, 0.1% Tween-20 at 4 °C with gentle shaking, overnight. Primary antibodies used were anti-α-tubulin (mouse monoclonal, Santa Cruz Biotechnology Inc., SC-8035), anti-pan-AKT (rabbit monoclonal, Cell Signalling Technology, 4691), anti-phospho-AKT (Ser473) (rabbit monoclonal antibody, Cell Signalling Technology, 4060), anti-phospho-AKT (Thr308) (rabbit monoclonal, Cell Signalling Technology, 13038), anti-p44/p42 (ERK1/2) 137F5 (rabbit monoclonal, Cell Signalling Technology, 4695), anti-phospho-p44/p42 MAPK (ERK1/2) (T202/Y204) (rabbit polyclonal, Cell Signalling Technologies, 9101), Rb (rabbit polyclonal, Santa Cruz Biotechnology, SC-7905) and pan-actin (rabbit polyclonal, Cell Signalling Technology, 4968). Horseradish peroxidase conjugated anti-mouse or anti-rabbit IgG (Santa Cruz Biotechnology Inc.) and Luminata Crescendo Western HRP substrate (EMD Millipore) were used for detection.

### Migration assay

Control scrambled or shRb LNCaP cells were conditioned with hypoxia (1% O_2_) or maintained at normoxic conditions and treated with either vehicle or drug (10 μM U0126, 2 μM A6730 or 10 nM INK128) for 96 h. After 96 h, cells were washed, trypsinized, counted on a Haemocytometer and then seeded on ThinCerts (Greiner Bio-One) with 8.0 μm pore size at 1 × 10^4^ cells per cell culture insert. The cells were maintained in serum-free RPMI 1640 with L-glutamine in the upper chamber whereas the lower chamber contained RPMI 1640 media with L-glutamine and 50% FBS. Cells were treated as stated above for a further 72 h and then total migrating cells were counted using an Olympus CKX41 light microscope.

### Immunofluoresence analysis and confocal microscopy

Rb proficient and deficient LNCaP cells were seeded on poly-L-lysine coated coverslips in six-well plates. The next day, cells were treated with either DMSO, 10 μM U0126 or 2 μM A6701 and then exposed to 1% O_2_ or left at normoxia for 72 hours. Cells were fixed with 4% paraformaldehyde in PBS and then permeabilized with 0.1% Triton X-100 in PBS. Cells were blocked with 1% BSA in PBS for 30 minutes and then incubated with TRITC-conjugated Rhodamine phalloidin (Life Technologies) for 1 hour at room temperature. Cells were washed with PBS, counter stained with 4′,6-diamidino-2-phenylindole (DAPI) and then mounted on slides with FluorSave (Calbiochem). Cell morphology was observed using a Zeiss LSM 780 confocal microscope and 63X objective lens magnification. Each image was recorded using the same power, magnification and exposure settings and cells were imaged using ZEN software (ZEISS Microscopy).

For the double-blind analysis, images consisting of ≥50 cells for each treatment condition were collected under the same power, exposure and magnification. Image files were then encrypted and a skilled person familiar with cellular structures but blind to the treatments and the expected results used ImageJ software (V1.51k, National Institutes of Health, Bethesda, Maryland, USA) to analyze fluorescent measurements of cell-cell boundaries and filopodia-like actin processes. All RGB images were first converted to 8-bit gray scale images for the imageJ analysis. To analyze the actin fluorescence in cell-cell boundaries and filopodia-like processes, the Freehand tool was used to carefully enclose these structures in each image. Then, the minimum, the maximum, and the average fluorescent intensity within each defined area were recorded. The detailed measurements for each image as well as a summary of the results for different treatments can be found in the Supplementary Dataset Files 1 and 2 (“Double-Blind Measurements of Individual Images.xlsx” and “Double-Blind Summary of Each Treatment.xlsx” respectively).

### Oscillating optical tweezer mechanical characterization

The stiffness of shRNA LNCaP cells was measured using an oscillating OT as described previously^26^. Briefly, the OT experimental set up is composed of a 3 W continuous wave, Nd:YAG laser beam with a wavelength of 1064 nm. The Nikon TE2000 inverted microscope is employed and the laser beam is highly focused at the sample surface by a 100X objective. The tripeptide Arg-Gly-Asp (RGD)-coated microbeads bound to the cell surface were trapped by the laser tweezer and then moved back and forth via oscillating laser trap with various frequencies of 0.1, 1, 10 Hz. As the bead moves sinusoidally, the cell develops internal stress and resistance to the bead motion that depends on the cell’s mechanical properties. The resultant bead displacement in response to the force applied by the tweezer was measured optically and the bead motion equation was used to calculate the cell shear modulus and viscosity in the frequency domain. The NARBM is the normalized representation of the measured microbead’s displacement (x(t)) due to applied force and is described by equation (1):

1 $${\rm{NARBM}}=\frac{x(t)}{{r}_{cont}},$$

where r_cont_ is the bead-cell contact radius (0.25~0.45 *μm*). More details about r_cont_ can be found in ref. 26.

The cell relaxation time can also be measured by a microbead linked to a cell in viscous solution trapped by the OT. Movement of the trapped bead due to viscous drag force of the surrounding solution as well as the thermal noise of the laser causes a cell to develop an internal stress that also depends on the cell’s mechanical properties. The bead motion in response to such forces is measured and by performing autocorrelation analysis on the bead position signal, we can find the cell relaxation time as derived below.

### Data Analysis

In order to analyze the experimental data and calculate the changes in cell mechanical properties, a bead motion equation was used as described previously^26^. The cell relaxation time was calculated by measuring fluctuations in the position of trapped beads linked to the cell cytoskeleton. By analyzing the normalized position auto-correlation function of trapped bead signal fluctuations in time domain, relaxation time was determined. The trapped bead motion equation can be described by equation (2):

2 $$x^{\prime} +(1/{\tau }_{0})x=\xi (t),$$

$${\tau }_{0}=\frac{(\gamma +\beta )}{{k}_{1}}$$

$$\gamma =6\pi a{\eta }_{med}$$

$$\beta ={r}_{cont}{\eta }_{cell}$$

where *x* is position of fluctuating trapped bead, *a* is the bead radius (1.5 *μm*), *η*_*med*_ is viscosity of surrounding media (10^−3^ *Pas*), and *ξ*(*t*) is thermal white noise. *η*_*med*_ and *η*_*cell*_ are the viscosity coefficients of the surrounding medium and the cell structure, and *k*_1_ is the laser stiffness coefficient, and *τ*_0_ is the relaxation time. Integrating both sides of the above equation with respect to time, we arrived at equation (3):

3 $$x(t)={e}^{-t/{\tau }_{0}}{\int }_{-\infty }^{t}\frac{1}{(\gamma +\beta )}\xi (\tau ^{\prime} ){e}^{\frac{\tau ^{\prime} }{{\tau }_{0}}}d\tau ^{\prime} ,$$

Calculating the normalized position autocorrelation function, and using the statistical properties of *ξ*(*t*) which are described according to the Einstein diffusion equation, we arrived at equation (4):

4 $$\begin{array}{rcl} < {\xi }_{i}(t) >  & = & 0\\  < {\xi }_{i}(t){\xi }_{j}(t^{\prime} ) >  & = & 2\gamma \beta {k}_{B}T\delta (t-t^{\prime} )\\  < {x}_{i}(t){x}_{i}(t+\tau ) >  & = & {e}^{\frac{-\tau }{{\tau }_{0}}}\end{array},$$

where *k*_*B*_ is Boltzmann constant (1.3806 × 10^−23^ *m* ^2^ *kgs* ^−2^ *K* ^−1^), and *T* is Room temperature (300 K).

Thus, by performing autocorrelation analysis on bead fluctuations and fitting the experimental results to the exponential function, we can determine the cell relaxation time

### Statistical analysis

For the optical tweezer experiments, qPCR and migration assays, all data are represented as means ± standard deviation (S.D.) and we used an ANOVA test for comparing more than two groups of samples’ means for statistical analysis. *P* < *0*.*05* was considered statistically significant. For the double blind analysis, we used a Kruskall-Wallis analysis with Dunn’s multiple comparisons test (alpha = 0.05; P-value < 0.05) to analyze mean pixel intensities along cell-cell boundaries. Statistical significance of the proportion of cells displaying filopodia-like clusters in each treatment group was determined using two-sided Fisher’s exact tests. All statistical analysis was conducted using GraphPad Prism software (GraphPad Software, Inc.).

## Electronic supplementary material

Supplementary Information

Dataset 1

Dataset 2
