# Supplementary material for: Retinoblastoma protein (Rb) links hypoxia to altered mechanical properties in cancer cells as measured by an optical tweezer
Source: Sci Rep. 2017 Aug 10;7:7833. doi: 10.1038/s41598-017-07947-6 (PMC5552853; doi:10.1038/s41598-017-07947-6)
Supplement: Supplementary file 1 — Supplementary Information [file 41598_2017_7947_MOESM1_ESM.pdf]

Supplementary Information 1

Retinoblastoma protein (Rb) links hypoxia to altered mechanical properties in cancer cells as measured by an optical tweezer

Khakshour, S.; Labrecque, M.P,; Esmaeilsabzali, H.; Lee, F.J.S.; Cox, M.E.; Park, E. J.; and Beischlag, T. V.

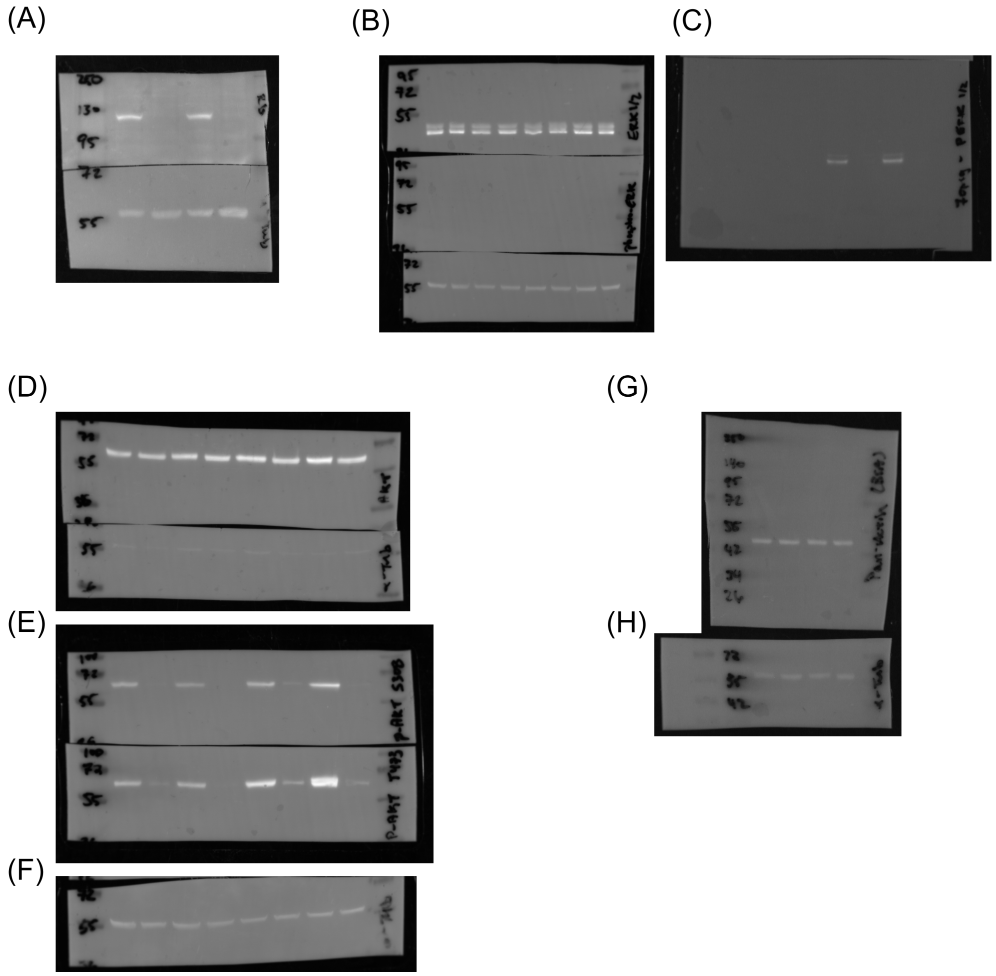

Figure 1. Complete blot images used for the main figures in the manuscript. Images A-H are composite images derived from the white light exposure overlaid with the chemiluminescent exposure used in the main figures. Composite images are shown to ensure visualization of molecular weight markers and/or complete blot outlines. **(A)** Blot was probed with primary antibodies to Rb (top bands) and α-tubulin (bottom bands) and represent the blot shown in Figure 1A. **(B)** and **(C)** Represent blots shown in Figure 3C. Blots in (B) are probed with primary antibodies to total ERK (top bands), α-tubulin (bottom bands) and pERK (T202/Y204). The pERK (T202/Y204) primary antibody used in (B) was not functional so a different pERK (T202/Y204) primary antibody was used in (C) on samples from the same experiment run in parallel. **(D)-(F)** Represent blots shown in Figure 4C. Blots in (D) were probed with primary antibodies to AKT (top) and α-

tubulin (bottom). Blots in (E) were probed with primary antibodies to phospho-AKT (Thr308) (top) and phospho-AKT (Ser473) (bottom). The blot in (F) was probed with a primary antibody α-tubulin. Gels for blots in (D) and (E) were loaded with 50ug total protein whereas (F) was loaded with 100ug total protein. Samples were derived from the same experiment and blots were processed in parallel. **(G)** and **(H)** Represent blots shown in Figure 6A and were probed with primary antibodies to pan-actin or α-tubulin respectively. Samples were derived from the same experiment and blots were processed in parallel.
